# Prevalence and antibiotic susceptibility patterns of uropathogens in men with prostate cancer and benign prostate hyperplasia from Southwestern Nigeria

**DOI:** 10.1186/s12866-024-03524-w

**Published:** 2024-09-21

**Authors:** Sharon O. Akinpelu, Grace I. Olasehinde, Stephen O. Ikuerowo, Olayemi O. Akinnola

**Affiliations:** 1 Department of Biological Sciences, College of Science and Technology, Covenant University, Ota, Nigeria; 2 Department of Surgery, Urology Division, Lagos State University College of Medicine, Ikeja, Nigeria

**Keywords:** Antibiotic sensitivity, *Enterobacteriaceae*, Inflammation, Prostate cancer, Urinary pathogens

## Abstract

**Background:**

Epidemiological investigations have revealed an important association between infection, inflammation and prostate cancer. Certain bacterial species, such as *Klebsiella* spp, *Escherichia coli*, *Pseudomonas* spp, *Proteus mirabilis*, *Chlamydia trachomatis* have been linked to prostate cancer. This study aimed to examine the microbiota; specifically bacterial species that have been linked to prostate infections in the urine of individuals diagnosed with prostate cancer.

**Results:**

Sixty-six prostate cancer patients and forty controls provided midstream urine samples. The urine samples were grown on suitable medium, and bacterial isolates were detected by standard microbiological methods. Additionally, the antibiotic sensitivity pattern of the bacterial isolates was analysed. A total of number of 72 bacterial isolates were obtained from the urine of study participants. The results showed the presence of *Escherichia coli* (50.0%), *Pseudomonas aeruginosa* (18.1%), *Klebsiella* spp (15.3%), *Staphylococcus aureus* (8.3%), *Enterobacter* spp (4.2%), and *Proteus mirabilis* (2.8%) in the urine. The most common bacterial species isolated from prostate cancer patients was *Escherichia coli*, which was susceptible to levofloxacin (100%), tobramycin (91.7%), and amikacin (62.5%).

**Conclusions:**

This study’s findings established the presence of bacteria previously linked to prostatitis. This report indicates a high prevalence of pro-inflammatory bacteria and uropathogens in the urinary tract of men diagnosed with prostate cancer.

## Background

Prostate cancer (PC) ranks fifth in cancer-related mortality globally and is the second most prevalent cancer in males. Age, race, and family history have all been linked to prostate cancer [1]. Prostate cancer is the commonest cancer diagnosed among Nigerian men [2]. An increasing body of research suggests that inflammation might have an impact on prostate carcinogenesis [3].

A possible cause of prostate tissue inflammation is infections [4]. Prostatitis caused by microbial exposure results in the production of reactive oxygen species and inflammatory cytokines. These immune mediators, in turn, encourage cellular growth and may play a role in cancer development [5]. Most studies conducted so far have demonstrated a connection between prostatitis, sexually transmitted diseases (STDs), and prostate cancer development. According to two meta-analyses, prostate inflammation caused by prostatitis or STDs is linked to an increased risk of prostate cancer [6, 7].

One of the causes of chronic prostatitis is bacteria entering the prostate when urine flows back into the prostatic duct in the peripheral zone. In a study conducted on mice, uropathogenic *Escherichia coli* were transurethrally inoculated into the prostate. This model resulted in severe inflammation and reactive hyperplasia [8, 9].

It is reasonable to propose that inflammatory infectious agents may have a role in prostate cancer development, given that the prostate is in a region vulnerable to microorganisms present in urine and during sexual activity [10]. To date, no singular microorganisms are known to contribute to prostate cancer. However, there are many different bacterial species known to cause prostatitis, including *Escherichia coli* and *Enterococcus* spp, as well as inflammatory bacteria like *Propionibacterium acnes* [11]. This highlights the substantial contribution of bacteria that originate from the urinary tract in prostate cancer development [12].

The aim of this study was to investigate the occurrence of uropathogens in individuals with prostate cancer and benign prostate hyperplasia and their susceptibility to antibiotics.

## Materials and methods

### Study design

This cross-sectional study was conducted on 106 males attending the Urology Clinics of Lagos State University Teaching Hospital, Ikeja and Federal Medical Centre, Abeokuta. Male patients 40 years and above with prostate cancer diagnosis served as cases while those with benign prostate hyperplasia (BPH) served as controls. The Health Research and Ethics Committee of both hospitals approved this study (approval numbers: LREC/06/10/1794 and FMCA/470/HREC/01/2022/06). Each participant provided written consent before participating in the study. Males with recent antibiotic use for any indication were excluded from this study.

### Specimen collection and processing

Each study participant provided a 30 ml sample of clean–catch midstream urine, which was obtained in a sterile container with a screw cap. The specimen was properly labelled and transported to the laboratory to be processed. To analyse the urine, a loopful (0.001mL) was streaked onto the surface of MacConkey agar and blood agar plates and incubated aerobically at 37 °C for 24 h. Following incubation, any isolated colonies were subcultured onto nutrient agar and incubated at 37 °C for an additional 24 h. The resulting pure colonies were Gram stained and subjected to biochemical tests using Microbact Identification System (Oxoid, United Kingdom) to identify each isolate.

### Antibiotic susceptibility testing

Testing for antimicrobial susceptibility was done on bacterial isolates whose identity had been confirmed. The modified Kirby-Bauer disc diffusion technique was employed to carry out this experiment on Mueller Hinton agar plates following the guidelines established by the Clinical and Laboratory Standards Institute (CLSI, 2021) for various antibiotics including Trimethoprim/sulfamethoxazole (25 µg), ciprofloxacin (5 µg), tetracycline (30 µg), cefotaxime (30 µg), nitrofurantoin (300 µg), tobramycin (30 µg), levofloxacin (5 µg), erythromycin (15 µg), amikacin (30 µg), ceftriaxone (30 µg), sulfamethoxazole (25 µg), oxacillin (1 µg) and amoxicillin-clavulanic acid (30/10 µg) (Oxoid, United Kingdom).

### Multiple antibiotic resistance (MAR) index calculation

The MAR index is an economical and dependable technique for tracking the origins of antibiotic-resistant organisms [13]. The calculation of the MAR index value for an isolate involves determining the proportion of antibiotics to which the isolate is resistant, about the overall number of antibiotics administered. This value is an indicator of the isolates’ resistance profile [14]. The formula for calculating the MAR index is as follows:

MAR index of an isolate = number of antibiotics resistant to/ number of antibiotics used.

### Statistical analysis

Data analysis was conducted with the Statistical Package for Social Sciences (SPSS) 24.0. Mean ± standard deviation, frequencies, and percentages were used to express data. *p* < 0.05 was considered significant.

## Results

### Demographic characteristics of study participants

In this study, a total of 66 (62.3%) prostate cancer patients and 40 (37.7%) males with benign prostate hyperplasia were enrolled. Study participants had an average age of 67.6 (SD ± 9.30) years. The characteristics of the study participants can be found in Table 1.

**Table 1:** Characteristics of study participants

Age group	PC *N* (%)	BPH *N* (%)	*P*-value
41–50	2 (3.0%)	3 (7.5%)	0.269
51–60	10 (15.2%)	7 (17.5%)	
61–70	24 (36.4%)	20 (50.0%)	
71–80	21 (31.8%)	9 (22.5%)	
81–90	7 (10.6%)	1 (2.5%)	
91–100	2 (3.0%)	0 (0.0%)	
Total	66 (100.0%)	40 (100.0%)	

### Distribution of uropathogens in study subjects

A total of 63 (59.4%) out of 106 study participants had bacteriuria. Uropathogens were more commonly isolated in patients with prostate cancer than controls; 42/66 (63.6%) vs. 21/40 (52.5%). A higher number of bacterial isolates was identified in prostate cancer patients, compared to the controls; 49/72 (68.1%) vs. 23/72 (31.9%). Gram-negative bacteria (87.8%) were more commonly isolated than Gram-positive bacteria (12.2%). The urine samples of the study participants revealed that *Escherichia coli* was the most commonly found isolate, accounting for 50.0%. This was followed by *Pseudomonas aeruginosa* (18.0%), *Klebsiella* spp (15.3%), *Staphylococcus aureus* (8.3%), *Enterobacter* spp (4.2%), and *Proteus mirabilis* (2.8%) (Table 2).

**Table 2:** Distribution of isolated uropathogens in prostate cancer (PC) and benign prostate hyperplasia (BPH)

Organisms	PC *N* (%)	BPH *N* (%)	Total *N* (%)	*P*-value
*Escherichia coli*	24 (48.9%)	12 (52.2%)	36 (50.0%)	0.729
*Pseudomonas aeruginosa*	7 (14.3%)	6 (26.1%)	13 (18.0%)	
*Klebsiella* spp	9 (18.4%)	2 (8.7%)	11 (15.3%)	
*Enterobacter* spp	2 (4.1%)	1 (4.3%)	3 (4.2%)	
*Staphylococcus aureus*	4 (8.2%)	2 (8.7%)	6 (8.3%)	
*Proteus mirabilis*	2 (4.1%)	0 (0%)	2 (2.8%)	
Beta-haemolytic *Streptococcus*	1 (2.0%)	0 (0%)	1 (1.4%)	

### Antibiotic susceptibility pattern of isolated uropathogens

*E. coli* was the most isolated bacterium from PC patients and showed a higher level of susceptibility to levofloxacin (100.0%), tobramycin (91.7%), and amikacin (62.5%) but no susceptibility to cefotaxime, amoxicillin-clavulanic acid, erythromycin, ceftriaxone and sulfamethoxazole (0.0%). A significant difference was found in the antimicrobial susceptibility profiles of *E. coli* between PC and BPH (0.001). Most Gram-negative bacteria isolated from prostate cancer patients and those with benign prostate hyperplasia were sensitive to levofloxacin, tobramycin, amikacin and tetracycline. The results of the antimicrobial susceptibility pattern of each bacterial species are shown in Table 3. Table 4 highlights the multidrug–resistance (MDR), extensively drug resistance (XDR) and pan drug resistance patterns of the bacterial isolates from men with prostate cancer and benign prostate hyperplasia.

**Table 3:** Antimicrobial sensitivity pattern of bacteria isolated from men with prostate cancer (PC) and benign prostate hyperplasia (BPH)

Bacteria	Group	Total *N*	SXT	CTX	CIP	TOB	TE	NIT	AUG	OX	LEVO	ERY	AMK	CRO	SULP	*P*-value
*E. coli*	PC	24	12.5	0.0	8.3	91.7	29.2	37.5	0.0	ND	100.0	0.0	62.5	0.0	0.0	0.001
	BPH	12	16.7	25.0	16.7	58.3	50.0	66.7	0.0	ND	0.0	0.0	0.0	0.0	0.0	
*P. aeruginosa*	PC	7	71.4	0.0	0.0	100.0	57.1	100.0	0.0	ND	100.0	14.3	42.9	14.3	0.0	0.837
	BPH	6	16.7	0.0	0.0	50.0	33.3	83.3	0.0	ND	0.0	0.0	0.0	0.0	0.0	
*Klebsiella* spp	PC	9	33.3	0.0	0.0	55.6	55.6	0.0	0.0	ND	100.0	0.0	88.9	0.0	0.0	0.348
	BPH	2	0.0	0.0	0.0	100.0	50.0	50.0	0.0	ND	0.0	0.0	0.0	0.0	0.0	
*Enterobacter* spp	PC	2	0.0	0.0	50.0	100.0	100.0	50.0	0.0	ND	0.0	0.0	0.0	0.0	0.0	0.997
	BPH	1	100.0	0.0	100.0	100.0	100.0	0.0	0.0	ND	0.0	0.0	0.0	0.0	0.0	
*S. aureus*	PC	4	25.0	0.0	50.0	100.0	100.0	50.0	75.0	0.0	100.0	50.0	100.0	0.0	0.0	0.854
	BPH	2	0.0	0.0	*5*0.0	100.0	*50*.0	100.0	*5*0.0	0.0	0.0	0.0	0.0	0.0	0.0	
*P. mirabilis*	PC	2	50.0	0.0	0.0	0.0	0.0	0.0	0.0	ND	100.0	0.0	100.0	0.0	0.0	
	BPH	0	0.0	0.0	0.0	0.0	0.0	0.0	0.0	0.0	0.0	0.0	0.0	0.0	0.0	
*Streptococcus* spp	PC	1	100.0	0.0	100.0	100.0	100.0	0.0	0.0	0.0	0.0	0.0	0.0	0.0	0.0	
	BPH	0	0.0	0.0	0.0	0.0	0.0	0.0	0.0	0.0	0.0	0.0	0.0	0.0	0.0	

SXT: Trimethoprim/sulfamethoxazole, CTX: cefotaxime, CIP: ciprofloxacin, TOB: tobramycin, TE: tetracycline, NIT: nitrofurantoin, AUG: amoxicillin-clavulanic acid, OX: oxacillin, LEVO: levofloxacin, ERY: Erythromycin, AMK: Amikacin, CRO: Ceftriaxone, SULP: sulfamethoxazole

**Table 4:** Distribution of multidrug-resistant (MDR), extensively drug resistant (XDR) and pan drug resistant bacteria from men with PC and BPH

Bacterial species	Group	MDR *N* (%)	XDR *N* (%)	PDR *N* (%)
*E. coli*	PC	24 (66.7%)	20 (76.9%)	0 (0.0%)
	BPH	12 (33.3%)	6 (23.1%)	0 (0.0%)
*P. aeruginosa*	PC	7 (53.8%)	3 (42.9%)	0 (0.0%)
	BPH	6 (46.2%)	4 (57.1%)	0 (0.0%)
*Klebsiella* spp	PC	9 (81.8%)	6 (85.7%)	1 (100.0%)
	BPH	2 (18.2%)	1 (14.3%)	0 (0.0%)
*Enterobacter* spp	PC	2 (66.7%)	0 (0.0%)	0 (0.0%)
	BPH	1 (33.3%)	0 (0.0%)	0 (0.0%)
*S. aureus*	PC	3 (60.0%)	0 (0.0%)	0 (0.0%)
	BPH	2 (40.0%)	1 (100.0%)	0 (0.0%)
*P. mirabilis*	PC	2 (100.0%)	1 (100.0%)	0 (0.0%)
	BPH	0 (0.0%)	0 (0.0%)	0 (0.0%)
*Streptococcus* spp	PC	1 (100.0%)	0 (0.0%)	0 (0.0%)
	BPH	0 (0.0%)	0 (0.0%)	0 (0.0%)

### Multiple antibiotic resistance (MAR) index

Isolated exhibited resistance to various classes of antibiotics. The MAR indices of the current isolates ranged between 0.4 and 1.0; so, the closer to one, the higher the resistance. All the isolated bacteria displayed MAR values exceeding the threshold value of 0.2. This indicates high exposure to antibiotics in their environment. The mean MAR indices ranged from 0.46 (*Staphylococcus aureus*) to 0.76 (*Escherichia coli*) (Table 5).

**Table 5:** Mean multiple antibiotic resistance (MAR) indices of isolated uropathogens

S/N	Bacterial isolates	MAR Index	BPH	*P*-value
		PC		
1	*Escherichia coli*	0.76	0.67	0.472
2	*Pseudomonas aeruginosa*	0.63	0.73	
3	*Klebsiella* spp	0.74	0.71	
4	*Enterobacter* spp	0.59	0.42	
5	*Staphylococcus aureus*	0.46	0.50	
6	*Proteus mirabilis*	0.67	-	
7	*Streptococcus* spp	0.54	-	

## Discussion

In this study, the prevalence of bacteriuria in patients with prostate cancer (PC) was found to be 40.6% (43/106), which is in line with the findings of Heidler et al. (46.5%) [15]. The risk of developing bacteriuria is high in these groups of individuals due to urinary stasis, poor bladder emptying, and urethral instrumentation such as catheterization and cystoscopy. Moreover, age-related decreases in zinc-associated antimicrobial factors and an increase in prostatic fluid alkalinity can affect urinary tract bacterial colonization [16, 17]. Additionally, a higher proportion of bacterial isolates was identified in prostate cancer patients, compared to the controls; 49/72 (68.1%) vs. 23/72 (31.9%). *Escherichia coli* and *Enterococcus* spp have been found in substantially higher levels in the semen and prostatic secretions of prostate cancer patients compared to those with benign prostate hyperplasia in another study [18].

*Escherichia coli* was the primary uropathogen identified in this study, which aligns with reports from other studies. This bacterium is commonly associated with both asymptomatic bacteriuria and symptomatic urinary tract infection (UTI) [19]. Uropathogenic *E. coli* is known to cause acute and chronic bacterial prostatitis and can cause DNA damage in the prostate [5].

Other uropathogens isolated in this study include *Pseudomonas aeruginosa*, *Enterobacter* spp, *Klebsiella* spp, and *Proteus mirabilis*. Acute prostate infections are often predominated by uropathogenic *E. coli* strains, *Klebsiella* spp, *Enterococcus*, *Pseudomonas*, and *Proteus* spp. The urinary tract is recognized as a potential source of inflammatory triggers for the prostate. Bacteria can invade the prostate gland by traveling up the urethra or by urine flowing back into the prostatic duct. Many strains of these uropathogens are capable of biofilm formation and exhibit resistance to multiple antibiotics [20]. It is difficult to treat infections of the prostate gland because medications do not penetrate the prostate tissue sufficiently. This can lead to persistent inflammation in the prostate, potentially causing the formation of preneoplastic lesions [4]. The identification of the causative agents responsible for bacterial prostatitis and the early treatment of the infection may be crucial in preventing prostate cancer. The prevalence of gram-negative bacteria as a primary cause of urinary tract infections in patients with benign prostatic hyperplasia and prostate cancer may inform the selection of appropriate empirical antimicrobial therapy for these individuals.

An earlier investigation of UTIs in Nigeria reported a 16.7% prevalence of *Klebsiella* spp [21]. The two most frequently isolated bacteria in this study were *Escherichia coli* and *Klebsiella* spp. Other studies reported a percentage occurrence of 11.7% and 18.3% in *Pseudomonas aeruginosa* [22, 23]. Delcaru et al. [24] found *Enterobacter* spp and *Proteus* spp in urinary tract infections of older individuals with prostate disease [25]. *Staphylococcus aureus* was the most isolated Gram-positive bacteria in this study. It is worth noting that previous studies have also highlighted the increasing role of *S. aureus* as a cause of UTI [25, 26]. The most isolated microorganisms in men with benign prostate hyperplasia were *Escherichia coli*, *Klebsiella* spp, *Pseudomonas aeruginosa*, and *Staphylococcus aureus* in this study. Benign prostatic hyperplasia is an important risk factor for bacteriuria and urinary tract infections [27].

The isolates showed a varied degree of susceptibility to the antibiotics tested. In comparison to the other antibiotics, the bacterial isolates exhibited greater susceptibility to levofloxacin, tobramycin, and amikacin compared to the other antibiotics. Kanu et al. [28] and Nwokolo et al. [29] also reported high sensitivity of uropathogens to quinolones and aminoglycosides [28, 29]. This study adds more credence to the previously reported efficacy of levofloxacin against most bacteria. This is important to this study due to the characteristic ability of fluoroquinolones to penetrate extracellular fluids and cells ensuring it reaches the prostate [30, 31]. Susceptibility of isolates to tobramycin and amikacin were high which could be because of the infrequent prescription of these drugs in the treatment of UTI infections. This might also be because aminoglycosides are parenteral preparations used with much restriction [32].

The prevalence of strains resistant to common first-line oral antibiotics such as Trimethoprim/sulfamethoxazole, cephalosporins, and amoxicillin-clavulanic acid has increased worldwide, and specifically in Nigeria [33]. An earlier study of Nigerian prostate cancer patients showed resistance rates of 53.7 − 92.7% for cephalosporins [34]. In the current study, we found similar resistance patterns. The *S. aureus* isolates in the study exhibited 100% resistance to amoxicillin-clavulanate and oxacillin. *S. aureus* is known for the acquisition of resistance and persists in eluding control efforts [35].

All the MAR indices values for the isolates in our investigation were greater than 0.2, indicating that the isolates came from settings where antibiotics are either heavily used or contaminated [14]. The high MAR indices values found in this investigation would indicate that the isolates were subjected to antibiotic pressure. This may have been caused by the population in the research region misusing antibiotics. As this study suggests, it might eventually boost the development of multidrug resistance.

In addition, the presence and multidrug resistance of these uropathogens have implications for post-biopsy infections. The conventional approach for diagnosing prostate cancer is a prostate needle biopsy. However, the procedure can lead to infectious complications. One of the most frequent side effects following a prostate biopsy is urinary tract infection. The most frequently isolated pathogen from post-prostate biopsy infection is uropathogenic *Escherichia coli.* As a routine practice, antimicrobial prophylaxis is recommended to prevent these complications [36]. By reducing the incidence of post-biopsy infections, hospitalization rates, and morbidity and mortality following prostate biopsy can be decreased.

### Limitations

The methods of isolation used may preclude the discovery of organisms that require specialized media or are non-culturable. Another limitation of the study was the lack of a molecular assay, for instance, PCR, to confirm the identity of the isolates.

## Conclusions

Our findings demonstrate that proinflammatory bacteria and uropathogens are common in the urinary tract of prostate cancer patients. It also highlights the importance of investigating the antimicrobial susceptibility patterns of uropathogens to determine appropriate treatment for urinary tract infections in these patient groups.

## Data Availability

The datasets used and/or analysed during the current study are available from the corresponding author on reasonable request.
